# ﻿A new species of *Pseudophanerotoma* (Hymenoptera, Braconidae) from Nayarit, Mexico

**DOI:** 10.3897/zookeys.1095.74308

**Published:** 2022-04-14

**Authors:** Armando Falcón-Brindis, Jorge L. León-Cortés, Rubén F. Mancilla-Brindis, Mario Orlando Estrada-Virgen, Octavio J. Cambero-Campos

**Affiliations:** 1 Departamento de Conservación de La Biodiversidad, El Colegio de La Frontera Sur, María Carretera Panamericana y Periférico Sur, CP 29290, San Cristóbal de Las Casas, Chiapas, Mexico El Colegio de La Frontera Sur San Cristóbal de Las Casas Mexico; 2 Department of Entomology, University of Kentucky Research and Education Center, 348 University Drive, Princeton, KY 42445, USA University of Kentucky Research and Education Center Princeton United States of America; 3 Posgrado en Ciencias Biológico Agropecuarias, Unidad Académica de Agricultura, Universidad Autónoma de Nayarit, Carretera Tepic-Compostela Km 9, CP 63155, Xalisco, Nayarit, Mexico Universidad Autónoma de Nayarit Xalisco Mexico

**Keywords:** Cheloninae, COI barcode, integrative taxonomy, Mexican biodiversity, Neotropical region, parasitoid wasp, Tortricidae

## Abstract

Parasitoid wasps are known to be among the most abundant and species-rich on Earth and thus considered an ecologically important group of arthropods. Braconid wasps play a key role in regulating the populations of Lepidoptera, Coleoptera, and Diptera. However, the biology and taxonomy of numerous parasitoid species remain poorly known. In Mexico, only 17 species of the subfamily Cheloninae have been described. A new species of *Pseudophanerotoma* Zettel, 1990 (Hymenoptera, Braconidae), *P.huichol***sp. nov.**, is described from Nayarit, Mexico. The tortricid moth *Cryptaspasmaperseana* Gilligan & Brown, 2011 is reported as the host of this parasitoid wasp. Detailed taxonomic and barcoding information are provided.

## ﻿Introduction

The family Braconidae comprises 21,221 species worldwide (Yu et al. 2016), and the description of new species is exponentially increasing in the Neotropical region (Fernandez-Triana and Boudreault 2018; Sharkey et al. 2021). Braconid wasps are primarily specialized parasitoids of Lepidoptera, Coleoptera, and Diptera, thus playing an important role in regulating their populations. Usually, braconid wasp does not oviposit in the host egg (Wharton et al. 1997). However, members of the subfamily Cheloninae oviposit in the eggs of Lepidoptera and emerge from later larval instars or the pupa (Quicke 2015).

Cheloninae are mostly solitary endoparasitoid wasps of several Microlepidoptera, i.e., Pyraloidea and Tortricoidea (Shaw 1997). Within this subfamily, the genus *Pseudophanerotoma* Zettel, 1990 is highly specialized in parasitizing tortricid moths (Costa Lima 1956; Hoddle and Hoddle 2008; Penteado-Dias et al. 2008). Members of this genus are restricted to the Americas, occurring from southern Texas to Peru and Brazil (Zettel 1990; Kittel 2018; Sharkey et al. 2021).

One of the main limitations of studying parasitoid wasps is the large number of species and high variability in life histories (Shaw 1995; Hanson and Gauld 2006). The taxonomy of Braconidae has been poorly documented, especially in the tropics (LaSalle and Gauld 1991). Since the classical description of species started undergoing the so-called taxonomy crisis almost two decades ago (Godfray 2002), the integrative taxonomy framework (Dayrat 2005) has brought complementary approaches to tackle challenging groups. As in most parasitoid wasps, the taxonomy of Cheloninae has been changing and the use of molecular tools allowed advances for the systematics of the group. For instance, *Pseudophanerotoma* and *Furcidentia* Zettel, 1990 are now two separate genera based on molecular and morphological characters (Zettel 1990; Kittel et al. 2016). Kittel (2018) essentially cleared up the taxonomy of both genera and added new species, but a recent work also provided additional species names (Sharkey et al. 2021).

In Mexico, *Pseudophanerotoma* members correspond to a poorly documented taxon with a few records from the States of Oaxaca, Quintana Roo, Tamaulipas, Veracruz, and Yucatán (Sánchez-García and López-Martínez 2000; Coronado-Blanco 2011). However, none of these specimens have been determined to species level, limiting our knowledge upon such specialized genus but most importantly, reducing our ability to identifying priority biodiversity areas in the face of human disturbance (Ceballos et al. 2015). In this work, we describe a new species, *Pseudophanerotomahuichol* sp. nov., which attacks the tortricid moth *Cryptaspasmaperseana* Gilligan & Brown, 2011 occurring in Nayarit, Mexico (Mancilla-Brindis et al. 2019). We provide detailed taxonomic diagnoses for both sexes and support our findings with DNA barcoding. In addition, we present an updated checklist of *Pseudophanerotoma* species, their potential phylogenetic relationship, and discuss elements of their biology and distribution.

## ﻿Methods

As part of a survey to evaluate the prevalence of *Cryptaspasmaperseana* among avocado orchards in Nayarit, Mexico, several fruits and seeds were collected from December 2019 to October 2020 (Mancilla-Brindis et al. 2021). The rearing process was conducted at the Universidad Autónoma de Nayarit. The emerged wasps were preserved in 96% ethanol and shipped to the Collection of Entomology (**ECO-SC-E**) of El Colegio de la Frontera Sur (**ECOSUR**), San Cristóbal de las Casas, Chiapas, Mexico, for taxonomic identification.

DNA extraction was conducted at the Barcoding Laboratory of Life of ECOSUR, Chetumal, Quintana Roo, Mexico, using standard protocols (Hebert et al. 2003; Ivanova et al. 2006). Extracted DNA was amplified for a 650-bp region near the 5' terminus of the cytochrome c oxidase subunit I (COI) gene using the primers ZplankF1 (TCT ASW AAT CAT AAR GAT ATT GG) and ZplankR1 (TTC AGG RTG RCC RAA RAA TCA) (Posser et al. 2013). Amplified PCR product was then shipped to Eurofins Scientific, Louisville, KY, USA, for purification and sequencing using Sanger technology. Sequence was cleaned and aligned using BioEdit (Hall 1999). We performed a phylogenetic analysis using the neighbor-joining method (Saitou and Nei 1987), based on a bootstrap consensus tree inferred from 10,000 replicates (Felsenstein 1985) to evaluate the relationship of 10 *Pseudophanerotoma* species. In addition, a genetic distance matrix between pairs of sequences was calculated through maximum composite likelihood to estimate the evolutionary divergence between species (Tamura et al. 2004). *Pseudophanerotomaalvarengai* Zettel, 1990, *P.longicornia* Zettel, 1990, *P.maculosa* Zettel, 1990, *P.peruana* Zettel, 1990, and *P.zeteki* (Cushman) 1922 where not included since there is no barcoding information for them. The analysis involved 10 nucleotide sequences, eliminating positions with missing data. Bootstrap analysis, genetic distance matrix, and phylogenetic tree were conducted in MEGA11 (Tamura et al. 2021).

Morphological determination of *Pseudophanerotoma* followed Sharkey et al. (2021). Taxonomic features provided in this study followed van Achterberg (1988) and Wharton et al. (1997). Images were taken with a Leica MC 170 HD camera, adapted to a Leica M205C stereoscopic microscope. Morphometric data and image aligning was performed with the Leica Application Suite software. Holotype and paratype specimens are deposited in ECO-SC-E.

We used the following abbreviations to describe key ocellar measurements (Kittel 2018):

**LOL** lateral ocellar line (distance between lateral and anterior ocelli);

**OOL** ocular ocellar line (distance between lateral ocellus and compound eye);

**POL** posterior ocellar line (distance between lateral ocelli).

## ﻿Results

### ﻿ Pseudophanerotoma huichol

Taxon classificationAnimaliaHymenopteraBraconidae

Falcón-Brindis sp. nov.

88CE9D0E-E355-5256-AF7A-7AF0DF8FF3B7

http://zoobank.org/DB6DDDDA-E2C6-4DD6-9BCB-438870114206

#### Diagnosis.

*Pseudophanerotomahuichol* sp. nov. can be distinguished from the other species by the unicolorous head, meso, and metasoma, except for the dark brownish integument on the apical half of hind femora and a small spot on the mesopleuron (sometimes absent in males); antenna with 52 antennomeres in females and 46 antennomeres in males, occipital carina complete.

#### Description (female).

**Figure 1.**
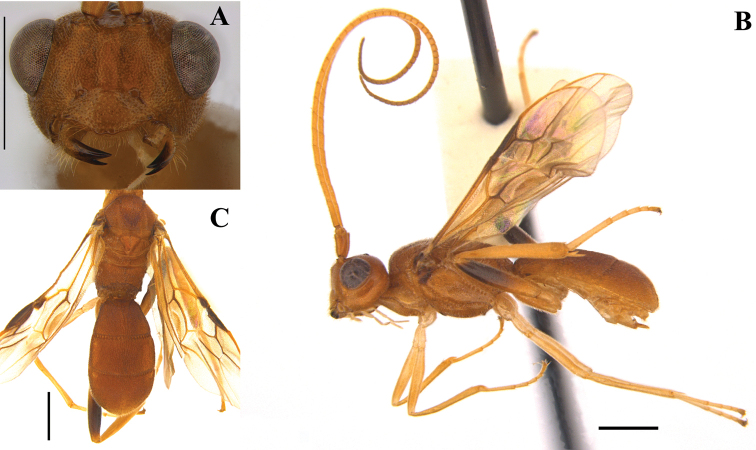
*Pseudophanerotomahuichol* sp. nov., female **A** head frontal **B** lateral habitus **C** dorsal view of meso and metasoma. Scale bars: 1.0 mm.

Body length 5.5 mm; ratio of length of fore wing to body 0.8; ratio of metasoma to mesosoma 1.2 (Fig. 1A–C).

***Head*.** Antenna with 52 antennomeres; ratio of width of face to its height in frontal view 1.4; ratio of width of clypeus to its height 1.8; ratio of length of third antennomere to width 3.7; ratio of length of fourth antennomere to width 3.0; ratio of length of penultimate antennomere to width 1.3; malar space to base of mandible 0.3 mm. Clypeus convex and sparsely punctate with two teeth; face straight in lateral view, punctate; frons and vertex densely punctate; ratio of LOL:POL:OOL 0.1:0.1:0.1:0.4.

**Figure 2.**
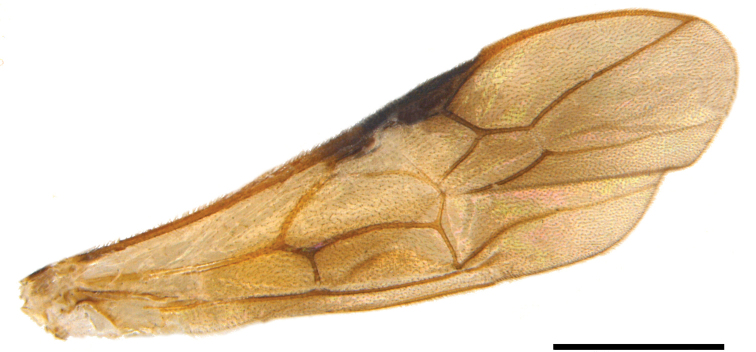
*Pseudophanerotomahuichol* sp. nov., fore wing. Scale bar: 1.0 mm.

***Mesosoma*.** Mesoscutum shiny and densely punctate, mid mesoscutal area coarsely sculptured; notauli present; mesopleuron punctures shallow and less dense; scutellar sulcus present, with coarse pits; mesoscutellum convex and punctate; propodeum areolate; propodeal tubercles absent; ratio of mesosoma height to its length 0.6; ratio of hind tibia to hind tarsus 2.1; ratio of length to width of hind coxa 2.0; ratio of length to width of hind femur 5.2; ratio of length to width of hind tibia 7.5; ratio of length to width of hind tarsus 6.2; ratio of length of fore wing to body 0.8; fore wing length 4.2 mm; 1RS present; M curved; RS+M spectral (Fig. 2).

***Metasoma*.** Oval in dorsal view; sculpture striate throughout; ratio of metasomal width to length 0.6; ratio of length of the three metasomal tergites 0.7:0.7:0.8.

***Color*.** Scape, head, meso and metasoma ferruginous; antennomeres and legs pale orange to yellowish; dark brown integument on tegula, a small spot on the top of mesopleuron (below the tegula) and apical half of hind femora. Wings hyaline with greenish-purplish reflections, wing venation brown to dark brown; parastigma and pterostigma dark brown.

#### Type material.

Holotype and paratypes specimens are pinned and deposited in ECO-SC-E. ***Holotype***: Mexico: ♀, Lo de Lamedo, Tepic, Nayarit 21.54222, –104.92833; 880 m elev., 10 Oct. 2020; 21.53388, –104.93722; 860 m elev., 04 Sep. 2020; R.F. Mancilla-Brindis leg. Holotype voucher code 69271. ***Paratypes***: 6 ♀: 69272, 69273, 69274, 69275, 69276, 69277; 5 ♂: 69278, 69279, 69280, 69281, 69282.

#### Description (male).

**Figure 3.**
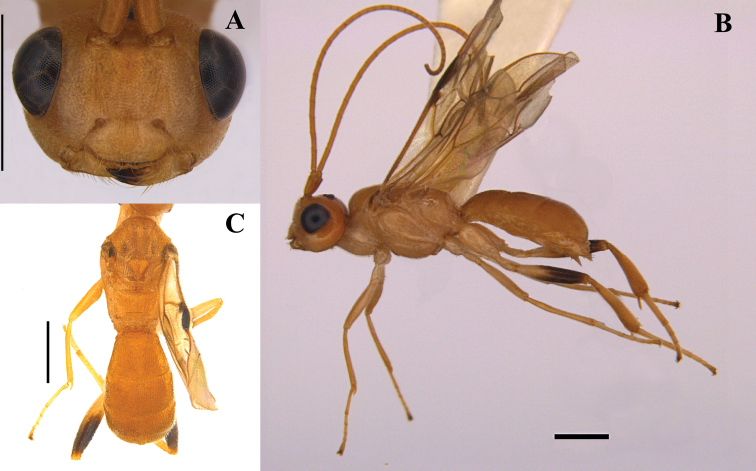
*Pseudophanerotomahuichol* sp. nov., male **A** head frontal **B** lateral habitus **C** dorsal view of meso and metasoma. Scale bars: 1.0 mm.

Antenna with 46 antennomeres. Body length 5.2 mm; ratio of length of fore wing to body 0.8; ratio of length of metasoma to mesosoma 1.2 (Fig. 3A–C).

***Head*.** Antenna with 46 antennomeres; ratio of width of face to its height in frontal view 1.2; ratio of width of clypeus to its height 2.0; ratio of length of third antennomere to width 4.5; ratio of length of fourth antennomere to width 3.6; ratio of length of penultimate antennomere to width 1.9; malar space to base of mandible 0.3 mm. Clypeus convex and sparsely punctate with two teeth; face straight in lateral view, punctate; frons and vertex densely punctate; ratio of LOL:POL:OOL 0.1:0.1:0.1:0.3.

***Mesosoma*.** Same sculpture patterns as in female; ratio of mesosoma height to its length 0.7; ratio of hind tibia to hind tarsus 1.9; ratio of length to width of hind coxa 2.1; ratio of length to width of hind femur 4.4; ratio of length to width of hind tibia 5.3; ratio of length to width of hind tarsus 5.8; ratio of length of fore wing to body 0.8; fore wing length 4.1 mm.

***Metasoma*.** Oval in dorsal view; sculpture striate throughout; ratio of metasomal width to length 0.6; ratio of length of the three metasomal tergites 0.6:0.6:0.8.

***Color*.** In general, male is paler than female; scape, head, meso and metasoma light orange; antennomeres and legs pale yellow to beige; dark brown integument on tegula and apical half of hind femora. Wings the same color pattern as in female.

#### Remarks.

This species differs from all the other congeners by having a large number of antennomeres in both sexes: 52 and 46 antennomeres in females and males respectively.

#### Biology.

Parasitoid of *Cryptaspasmaperseana*, a tortricid pest of avocado documented in Hidalgo, Michoacán, and Nayarit, Mexico (Mancilla-Brindis et al. 2019).

#### Sequence data.

GenBank accession number for this species is COIMZ501206.

#### Etymology.

The species is named in honor to the Huichol culture from Nayarit, Mexico.

**Figure 4.**
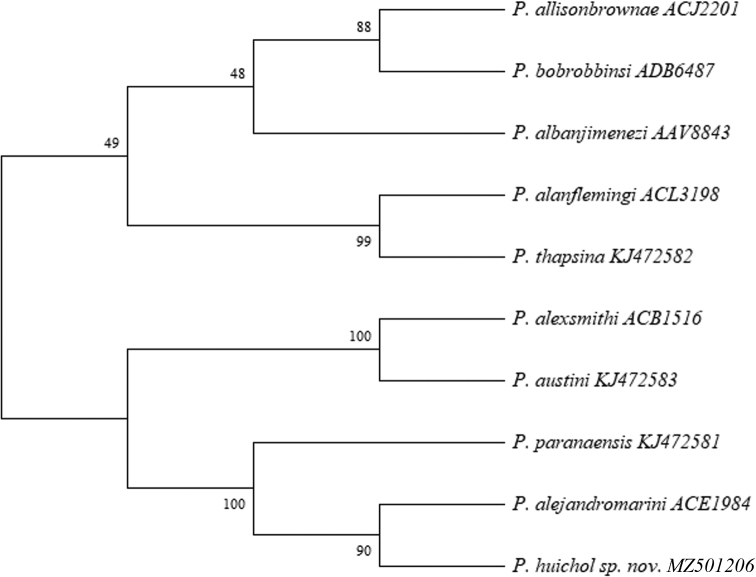
Bootstrap consensus tree of *Pseudophanerotoma* species. Five species are missing due to the lack of sequence information. The percentage of replicate trees in which the associated taxa clustered together in the bootstrap test are shown next to the branches. The lower clade has a weak bootstrap support.

According to the phylogenetic analysis for the barcoded *Pseudophanerotoma* species (Fig. 4), the closest species to *P.huichol* sp. nov. was *P.alejandromarini* Sharkey, 2021 (90%), both being sister species of *P.paranaensis* Costa Lima, 1956 (100%). The results from the evolutionary distance analysis showed that *P.alexsmithi* and *P.austini* are the closest species (0.0051), followed by *P.huichol* sp. nov. – *P.alejandromarini* (0.0133), *P.alejandromarini* – *P.paranaensis* (0.0261). In contrast, the pairwise comparison between *P.austini* – *P.allisonbrownae* and *P.alexsmithi* – *P.allisonbrownae* revealed the largest evolutionary divergence (0.2167) (Table 1).

**Table 1.** Estimates of evolutionary divergence between sequences. The number of base substitutions per site from between sequences are shown.

	*P.alanflemingi*	*P.albanjimenezi*	*P.alejandromarini*	*P.alexsmithi*	*P.allisonbrownae*	*P.bobrobbinsi*	*P.paranaensis*	*P.austini*	*P.thapsina*
*P.albanjimenezi*	0.1250								
*P.alejandromarini*	0.1188	0.1277							
*P.alexsmithi*	0.1186	0.1335	0.1120						
*P.allisonbrownae*	0.1843	0.1943	0.2052	0.2167					
*P.bobrobbinsi*	0.1442	0.1534	0.1412	0.1467	0.1663				
*P.paranaensis*	0.1248	0.1402	0.0261	0.1243	0.2085	0.1506			
*P.austini*	0.1124	0.1366	0.1088	0.0051	0.2167	0.1401	0.1210		
*P.thapsina*	0.0450	0.1371	0.1308	0.1278	0.2086	0.1634	0.1400	0.1214	
*P.huichol*	0.1220	0.1340	0.0103	0.1119	0.2089	0.1477	0.0314	0.1119	0.1341

## ﻿Discussion

This is the first work describing a new species of the genus *Pseudophanerotoma* in Mexico. Previous reports documented individuals at the generic level (Sánchez-García and López-Martínez 2000; Coronado-Blanco 2011). It is likely that *P.thapsina* (recorded in the United States and French Guiana) and *P.austini* (Guatemala) also occur in Mexico (Kittel 2018). However, further revisions are required to confirm their presence. Moreover, it is certain that other species of the genus occurring in Neotropical Mexico are deposited in museum collections (Sánchez-García and López-Martínez 2000; Coronado-Blanco 2011).

According to the most recent key for species of *Pseudophanerotoma* (Kittel 2018), *P.huichol* sp. nov., can be separated in couplet four, where the difference with *P.thapsina* is the dark brownish integument on the apical half of hind femora and the dark spot present on top of the mesopleuron. Despite the close phylogenetic relationship between *P.huichol* sp. nov. and *P.alejandromarini*, the latter species lacks dark integument on the hind femora. In summary, *P.huichol* sp. nov. has the largest number of antennomeres known from the species presented by Kittel (2018) and is undoubtedly different from those recently added by Sharkey et al. (2021).

Members of the subfamily Cheloninae have been used in the biocontrol of exotic pests (Narendran 2001). In Mexico, 704 species of Braconidae have been listed from 34 subfamilies (Coronado-Blanco et al. 2012), of which, only 17 species of Cheloninae have been identified (Coronado-Blanco and Zaldívar-Riverón 2014). However, most of these species belong to the genus *Chelonus* Panzer, 1806 (Coronado-Blanco et al. 2004), which is well known to control the populations of the fall armyworm *Spodopterafrugiperda* (J.E. Smith, 1797) feeding on forage maize in north and central Mexico (Rios-Velasco et al. 2011; Estrada-Virgen et al. 2013; García-González et al. 2020). Nonetheless, the biology of most chelonines occurring in Mexico remains unknown.

Likewise, the biology of the 15 known *Pseudophanerotoma* species has been poorly documented (Table 2). In most cases (66%), there is no information about host preferences and the same proportion is known from a single sex. The described *P.huichol* sp. nov. corresponds to the *Pseudophanerotoma* sp. reported parasitizing *C.perseana* in two localities from Nayarit, Mexico (Mancilla-Brindis et al. 2019, 2021). Therefore, this study has helped to identify an important interaction between *P.huichol* sp. nov. and a serious pest of avocado. However, further research is needed to provide details of, for example, the life history, distribution, and management of *P.huichol* sp. nov., potentially regulating the populations of *C.perseana*.

**Table 2.** A checklist of *Pseudophanerotoma* species according to Zettel (1990), Penteado-Dias et al. (2008), Kittel (2018), Sharkey et al. (2021), and this work. Only accepted scientific names are included. Types refer to the known sexes on each species.

Species	Occurrence	Host data	Types	Sequence accession number
*P.alanflemingi* Sharkey, 2021	Guanacaste, Costa Rica	Unknown	♀	BOLD:ACL3198
*P.albanjimenezi* Sharkey, 2021	Alajuela, Costa Rica	*Episimusortygia* (Tortricidae) feeding on *Vismiabaccifera* (Hypericaceae)	♂	BOLD:AAV8843
*P.alejandromarini* Sharkey, 2021	Guanacaste, Costa Rica	Unknown	♀	BOLD:ACE1984
*P.alexsmithi* Sharkey, 2021	Alajuela, Costa Rica	Cosmorrhyncha albistrigulana (Tortricidae) feeding on Dialium guianense (Fabaceae)	♀	BOLD:ACB1516
*P.allisonbrownae* Sharkey, 2021	Guanacaste, Costa Rica	Unknown	♂	BOLD:ACJ2201
*P.alvarengai* Zettel, 1990	Bahia, São Paulo, Brazil	Cydia tonosticha (Tortricidae) feeding on Stryphnodendron adstringens (Fabaceae)	♀♂	NA
*P.austini* Kittel, 2018	Petén, Guatemala	Unknown	♂	GenBank KJ472583
*P.bobrobbinsi* Sharkey, 2021	Guanacaste, Costa Rica	Unknown	♀	BOLD:ADB6487
*P.huichol* Falcón-Brindis, sp. nov.	Nayarit, Mexico	*C.perseana* (Tortricidae) feeding on *Perseaamericana* var. *Drymifolia* (Lauraceae)	♀♂	MZ501206
*P.longicornia* Zettel, 1990	Paraguay; Ecuador	Unknown	♀♂	NA
*P.maculosa* Zettel, 1990	Barro Colorado, Panama	Unknown	♂	NA
*P.paranaensis* Costa Lima, 1956	Paraná, Brazil; Saül, French Guiana	Olethreutes anthracana (Tortricidae)	♂	GenBank KJ472581
*P.peruana* Zettel, 1990	Alto-Samboroi, Peru	Unknown	♂	NA
*P.thapsina* Walley, 1951	Texas, California, Arizona, Florida, USA; Mt. Chevoux, French Guiana	Unknown	♀♂	GenBank KJ472582
*P.zeteki* (Cushman, 1922)	St. Bernardino, Panama	Unknown	♀♂	NA

Our preliminary phylogenetic analysis indicates that there is strong evidence for *P.huichol* sp. nov., *P.alejandromarini*, and *P.paranaensis* being sister species, albeit the cluster classification showed poor resolution (weak bootstrap support). In addition, the weak evolutionary divergence between *P.alexsmithi* and *P.austini* (0.0051) reveals the closest relationship among the genus *Pseudophanerotoma*. Such clades are naturally affected by the missing barcoded species, but the explanations could be attributed to several factors (e.g., lack of alternative barcoded regions, different gene history, or early diverging lineages) (Doyle 1992). Thus, our phylogenetic analysis should be interpreted with caution, as it only attempted to compare the position of the new species within the sequenced congeners.

Even though divergent opinions, molecular methods are now an important part of taxonomy, allowing integrative approaches (Padial et al. 2010). In this regard, many braconid species have been described using DNA barcoding (Smith et al. 2007, 2008; Sharkey et al. 2021). Sharkey et al. (2021) recently added more than 400 species to the list of Braconidae, including six new species of *Pseudophanerotoma*, with descriptions based on the COI barcoded region. This approach can certainly be helpful when describing large numbers of species, but morphological descriptions are handy when molecular tools are not available. In this sense, whereas the last taxonomic key to *Pseudophanerotoma* species did not thoroughly include molecular data (Kittel 2018), the six new species lack of morphological description (Sharkey et al. 2021) and thus further research is required to strengthen their presumed relationships.

## Supplementary Material

XML Treatment for Pseudophanerotoma huichol
